# Peroxisomes and Oxidative Stress: Their Implications in the Modulation of Cellular Immunity During Mycobacterial Infection

**DOI:** 10.3389/fmicb.2019.01121

**Published:** 2019-06-14

**Authors:** Geetanjali Ganguli, Utsav Mukherjee, Avinash Sonawane

**Affiliations:** ^1^School of Biotechnology, KIIT (deemed to be University), Bhubaneswar, India; ^2^Discipline of Biosciences and Biomedical Engineering, Indian Institute of Technology Indore, Indore, India

**Keywords:** mycobacteria, oxidative stress, peroxisomes, mitochondria, macrophages

## Abstract

Host redox dependent physiological responses play crucial roles in the determination of mycobacterial infection process. *Mtb* explores oxygen rich lung microenvironments to initiate infection process, however, later on the bacilli adapt to oxygen depleted conditions and become non-replicative and unresponsive toward anti-TB drugs to enter in the latency stage. *Mtb* is equipped with various sensory mechanisms and a battery of pro- and anti-oxidant enzymes to protect themselves from the host oxidative stress mechanisms. After host cell invasion, mycobacteria induces the expression of NADPH oxidase 2 (NOX2) to generate superoxide radicals (${\text{O}}_{2}^{-}$), which are then converted to more toxic hydrogen peroxide (H_2_O_2_) by superoxide dismutase (SOD) and subsequently reduced to water by catalase. However, the metabolic cascades and their key regulators associated with cellular redox homeostasis are poorly understood. Phagocytosed mycobacteria *en route* through different subcellular organelles, where the local environment generated during infection determines the outcome of disease. For a long time, mitochondria were considered as the key player in the redox regulation, however, accumulating evidences report vital role for peroxisomes in the maintenance of cellular redox equilibrium in eukaryotic cells. Deletion of peroxisome-associated peroxin genes impaired detoxification of reactive oxygen species and peroxisome turnover post-infection, thereby leading to altered synthesis of transcription factors, various cell-signaling cascades in favor of the bacilli. This review focuses on how mycobacteria would utilize host peroxisomes to alter redox balance and metabolic regulatory mechanisms to support infection process. Here, we discuss implications of peroxisome biogenesis in the modulation of host responses against mycobacterial infection.

## Introduction

According to the World Health Organization (WHO) report approximately 10.4 million global populations are infected with tuberculosis (TB) (WHO Global Tuberculosis Report 2017). Of which 64% of the new TB cases have been reported mainly in India followed by Indonesia, China, Nigeria, Pakistan, and South African countries. TB, caused by intracellular bacilli *Mycobacterium tuberculosis (Mtb*), affects individuals of all age groups primarily those with immune compromised system such as in human immunodeficiency virus (HIV) co-infected individuals. Another major challenge associated with TB disease is the emergence of multi-drug resistant (MDR) *Mtb* strains. Recent WHO report documented about 480,000 new MDR cases and 100,000 cases with rifampicin resistance (World Health Organization [WHO], 2017). Various factors contributed to the emergence of MDR *Mtb* strains such as inadequate TB treatment, longer treatment duration, patient’s non-compliance, and drug abuse. MDR-TB shows resistance against two most effective first-line drugs such as isoniazid (INH) and rifampicin (RIF). More recently cases of extremely drug resistance (XDR) and totally drug resistant (TDR) have been reported (Velayati et al., 2013). In XDR-TB, the bacilli shows resistance toward second line drugs (amikacin, kanamycin, capreomycin, and fluoroquinolones), in addition to INH or RIF; while TDR-TB is resistant to all first-line as well as second-line anti-TB drugs, and therefore is virtually untreatable. In addition, the only available live attenuated *M. bovis*-BCG vaccine has been proved ineffective to give protection in adult TB cases. Latent form of TB is another major concern as 90% of the infected individual exhibit a clinically “stand-off” condition as the bacilli resides in a favorable niche called “granuloma.” Studies showed that 5–10% of these latently infected cases can develop active TB during their life time. In spite of activation of both innate as well as adaptive immune responses, *Mtb* has the ability to impair proper antigen presentation to avoid recognition and killing of the bacilli (Pieters, 2008; Saini et al., 2014, 2016; Sreejit et al., 2014). TB still remains at the pinnacle among the infectious diseases. Thus to overcome these challenges, it is crucial to understand the basic molecular mechanisms of bacillary persistence and resistance in detail, which will lead to the development of effective treatment regimes by manipulating the host immune machinery.

After inhalation, macrophages act as the primary depots for the intracellular persistence of *Mtb* (Pieters, 2008), here the bacilli subvert host’s innate defense signaling cascades for persistence. *Mtb* aptly modulates the process of phago-lysosome biogenesis, which includes intermediate processes such as pathogen internalization, maturation of infected phagosomes, acidification of the phagocytic vacuole and finally phago-lysosome fusion. Immune cells such as macrophages, release ROS/RNS achieving intracellular killing of pathogens, however virulent mycobacteria by one way or other restrain this (Pieters, 2008; Ehrt and Schnappinger, 2009; Meena and Rajni, 2010; Saini et al., 2014; Lerner et al., 2015). The phago-lysosome fusion event is considered critical for proper antigen processing and presentation via major histocompatibility complex (MHC)- Class II molecules to T-cells. However, *Mtb* is known to block phago-lysosome fusion in order to promote its survival in macrophages (Lerner et al., 2015). It is well established that *Mtb* employs various other immune evasion strategies, however the molecular and cellular interplay between these events in poorly understood. Most of the drugs used for the treatment of TB infection primarily target the crucial enzymatic processes occurring in the bacteria; however, in order to develop a novel intervention approach it is equally important to augment host directed therapy. It is felt that manipulation of host oxidative stress molecules could be used effectively to manipulate signaling cascades to facilitate clearance of pathogens. Here, we will focus mainly on the role of various host receptors and organelles, which act as sites for redox balance during host–pathogen interaction.

## Modulation Of Macrophage Immune Effector Functions During Mycobacteria Infection

**FIGURE 1:**
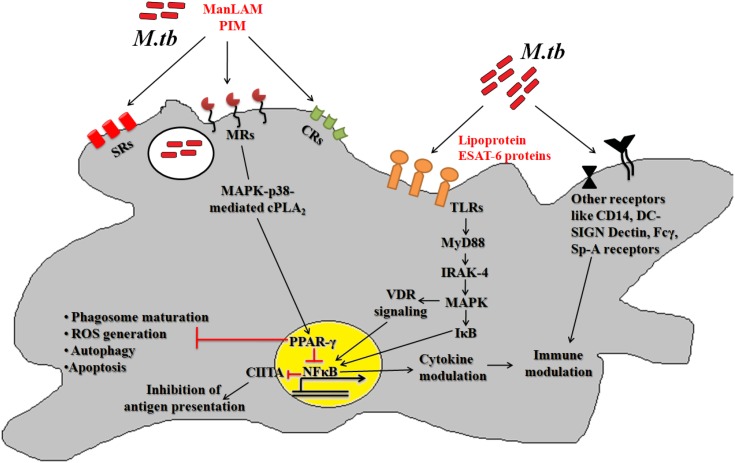
Host pathogen interaction: pathogenic *Mtb* enters the host via interaction between different host surface receptors like Toll like receptors (TLRs), mannose receptors (MRs), scavenger receptors (SRs), complement receptors (CRs) and so on and cognate *Mtb* ligands. These interactions modulates the phagosome maturation, downstream signaling molecules (like MYD88, IRAK-4, etc.), signaling pathways (like MAPK, autophagy, apoptosis, etc.) and transcription factors (like PPARG and NFκB) to favor bacillary persistence and reduced antigen presentation inside host cells.

After deposition into alveolar region, *Mtb* engages different cognate ligands to interact and invade alveolar macrophages. In this process, several virulence determinants such as cell surface proteins, enzymes and regulatory molecules of different metabolic pathways help *Mtb* to establish intracellular infection process. The *Mtb*-macrophage interaction involves participation of different pattern recognition receptors (PRRs), germline-encoded receptors of macrophages, and that of pathogen-associated molecular patterns (PAMPs) (Figure 1). Among the PPRs, toll-like receptors (TLRs) (TLR2 and TLR4), mannose receptors (MRs) and scavenger receptors (SRs) are known to play crucial roles during *Mtb* pathogenesis. Few reports suggested that TLRs also protect the host cells from mycobacterial infection via activation of nuclear factor kappa B (NF-κB) molecule and further downstream effector molecules and inflammatory cytokines (Sánchez et al., 2010; Basu et al., 2012). However, several *Mtb* lipoproteins or lipoglycans, encoded by *lpqH* (19-kDa lipoprotein) and the *lpr* gene family recognized by TLR2, TLR4, or TLR9 were shown to modulate cytokine production and signaling molecules like MYD88 and IRAK-4 to promote granuloma formation (Saini et al., 2014). In addition, *Mtb* secretory proteins such as early secretory antigenic target 6-kDa (ESAT-6) or several other ESAT-6 like proteins have been shown to interact directly with TLRs thereby alter the expression of interleukins (TNFA, IL12, IL27, IL1B) in infected macrophages. These proteins are also known to bind to beta-2-microglobulin (β2M) of MHC class-I molecules to block the antigen presentation (Sreejit et al., 2014). Recently, our group has shown that *Mtb* ESAT-6 family proteins *esxA* dampen macrophage immune responses, by inducing oxidative stress mediated genomic instability to promote mycobacterial persistence inside the host cells (Mohanty et al., 2016). Similarly MRs are expressed on the alternatively activated macrophages modulate the expression of ant-inflammatory cytokines after phagocytosis of *Mtb* (Rajaram et al., 2010, 2017; Stamm et al., 2015). The MR recognizes the terminal mannose, fucose or *N*-acetylglucosamine residues of mannosylated glycoproteins present on the *Mtb* cell wall. The interaction between MR and *Mtb* mannosylated proteins was found to inhibit or delay the phago-lysosome fusion process, thus allowing the bacteria to survive (Kang et al., 2005). The MR-mediated entry of *Mtb* is a relatively dynamic process in comparison to other PRRs. Because MR-*Mtb* association was found to instantaneously elicit the signaling cascades important for *Mtb* uptake (Rajaram et al., 2017). This interaction was also reported to modulate the synthesis of enzymes responsible for oxidative burst (Astarie-Dequeker et al., 1999). Recently, we observed that *Mtb* mannosylated phosphoribosyltransferase enzyme, encoded by *Rv3242c*, inhibits oxidative stress in infected macrophages as well as in adult zebra fish (Mohanty et al., 2015). Due to their pivotal role in early stages of disease, MRs are exploited for the treatment of diseases especially in cases where the infection sites cannot be accessed easily. In this context, targeting mannosylated agents such as coating of antibiotic-loaded liposomes with MR cognate ligands like sulfated sugar or mycobacterial mannosylated glycoproteins can be an attractive approach to reduce the jeopardy of TB disease development (Azad et al., 2014).

## Peroxisomal Nuclear Receptors Play Vital Role During *Mtb* Infection

Nuclear transcription factors or nuclear receptors (NRs) such as peroxisome proliferator-activated receptors (PPARs) are abundantly expressed in alternatively activated macrophages. These nuclear receptors exist in three isoforms such as PPARA, PPARB/D, and PPARG. PPARA interacts with the PPAR response component present on the promoter region of the target genes that are involved in the energy metabolism, mitochondrial and peroxisomal fatty acid oxidation (Tyagi et al., 2011). Recent study has demonstrated a crucial role for PPARA in the generation of immune responses against mycobacterial infection (Kim et al., 2017). It was observed that PPARA knock-out bone marrow derived macrophages (BMDM) failed to control the growth of *Mtb* and *M. bovis* BCG by modulating the NFκB signaling and pro-inflammatory cytokine production. Depletion of PPARA caused transcriptional repression of transcription factor EB (TFEB), which is important for the regulation of autophagic pathway (Kim et al., 2017; Taeg Oh et al., 2018). On the other hand the role of PPARB/D in bacterial infections is poorly understood. Its role has been mainly studied in the energy homeostasis and cellular metabolic processes (Wagner and Wagner, 2010; Palomer et al., 2018). The most extensively studied PPARG isoform, which is a ligand-dependent transcription factor expressed in alveolar macrophages, lymphocytes, and dendritic cells (Tyagi et al., 2011), plays an important role in immuno-regulation, energy and glucose metabolism, disease progression and pathology of bacterial infection (Reddy et al., 2016; Arnett et al., 2018). MR mediated *Mtb* entry was found to alter the expression of PPARG followed by trans-repression of different pro-inflammatory cytokines and transcription factors (NFκB, AP-1, STAT), TNFA, IL6, CXCL8, and COX2 enzyme production which are important in the generation of inflammatory responses through production of prostaglandins in macrophages (Rajaram et al., 2010). PPARG acts in response to stimulation by ligands such as thiazolidinediones (TZDs, which includes pioglitazone, rosiglitazone, troglitazone, ciglitazone), prostaglandins [for example 15-Deoxy prostaglandin J2, Prostaglandin A1 and D2, 9-hydroxyoctadecadienoic acid (HODE), 13-HODE], eicosanoids and lipids (Table 1). Ligand dependant activation of PPARG also plays a crucial role in the suppression of inflammatory response. PPARG ligand, TZDs, reduces the expression of TNFA, a key regulator of immune cell function. In addition rosiglitazone and ciglitazone treatment produced less TNFA, CXCL8 and IL6 thereby altered acute inflammation in mice (Kogiso et al., 2012; Kulkarni et al., 2012; Rajaram et al., 2017; Banno et al., 2018). Moreover, PPARG silencing reduced the bacterial count in CD11c^+^ cells isolated from *Mtb* infected mice lungs (Saini et al., 2018), indicating that transcription activation of PPARG is important in the determination of intracellular bacterial burden. Increased expression and nuclear localization of PPARG was observed upon infection with *M. bovis* BCG strain. The presence of active form of PPARG in the infected cells increased lipid body formation in resident alveolar macrophages (Mahajan et al., 2012). The presence of lipid bodies supports bacterial survival inside the host. Infection of Schwann cells with *M. leprae* also supported the importance of PPARG in mycobacterial replication and survival (Reddy et al., 2016). Altogether, these studies suggest that PPARG acts as a negative regulator of macrophage activation and also modulates the expression of different inflammatory genes and macrophage M1 to M2 polarization. Alternatively activated macrophages (or M2 macrophages) contribute to pathogenesis and immunoregulatory functions, thus are favored sites for persistent progression of infectious diseases. Like MR, PPARG is also abundantly expressed on the surface of alternatively activated macrophages, which exhibits anti-inflammatory activity to aid the intracellular *Mtb* growth (Rajaram et al., 2017). *N. caninum* infection induced PPARG dependent expression of MRC1, IL10, and other classical M2 macrophage markers (He et al., 2017). Thus downregulation of pro-inflammatory cytokines, nitrosative, and iron starvation stress in PPARG expressing M2 macrophages help in survival of pathogens rather than clearance (Kahnert et al., 2006; He et al., 2017). However, the underlying mechanistic insights are poorly understood. A recent study predicted a correlation between PPARG and macrophage apoptosis using NanoString database A. It is well-established that apoptosis is an immune defense mechanism to stop the intracellular bacterial growth. Thus targeted alteration in the expression of apoptotis related proteins may help in the control of bacillary proliferation. Expression of pro-apoptotic (like Bcl family members) and pro-survival markers (like MCL-1) were shown to be tightly regulated by the PPARG (Arnett et al., 2018). Latent *Mtb* infection is associated with formation of foamy macrophages and lipid rafts. This process is PPARG dependant, which further implicates that the intracellular bacilli utilize the host-derived metabolic pathways for its persistence. Further it has been reported that key immune metabolites of Vitamin D and B promote the inhibition of PPARG mediated lipid droplet formation thereby restricting the growth of mycobacteria (Bah et al., 2017; Hu et al., 2018). Vitamin B1 induces the transition of M2 to classically activated M1 macrophages thereby resulting in increased microbicidal microenvironment, TNFA and IL6 expression by limiting the expression of PPARG (Hu et al., 2018). Thus the pleiotropic effects of PPARG and its ligands/agonists on cellular metabolism during infection suggest that modulation of PPARG expression (and its ligands/agonists) alone or the interaction between MR and PPARG can be utilized as a promising host-directed therapy tool in the control of intracellular mycobacteria as well as disease progression.

**Table 1:** List of PPAR agonists/ligands and its related diseases.

PPAR ligands			Related diseases	Mode of action	References
PPARA	Agonist	Eupatilin	Degeneration of gastric mucosa	Cryoprotective effects against gastric mucosal damage by inducing anti-inflammatory and anti-oxidative phenotype	Ryoo et al., 2014; Jung et al., 2018
			Atopic dermatitis	Downregulates the expression of TNFA. IFNG and IL1B	
		Resveratrol	Obesity and metabolic syndrome	Increase in MUFA and PUFA	Castrejón-Tellez et al., 2016
		Fibrates	Hypertriglyceridemia Hypoalphalipoproteinemia	Downregulates hepatic apolipoprotein C-III thereby stimulating lipoprotein lipase gene	Botta et al., 2018
	Antagonist	NXT629	Chronic lymphocytic leukemia (CLL)	Inhibits PPARA agonists induced transcription of PPAR- α on CLL cells thereby inhibiting drug resistance and immunosuppressive property in the host	Messmer et al., 2015
PPARB/D	Agonists	GW501516 GW0742 L-165041	Obesity Type-2 diabetes Dyslipidemia Non-alcoholic fatty liver disease	Decreases subcutaneous and visceral adipose tissues Recovers glucose tolerance and insulin sensitivity Elevates lipid catabolism Introduces and equilibrium between pro- and anti-inflammatory molecules thereby reducing liver damage and inflammation	Girroir et al., 2008; Coll et al., 2009; Chen et al., 2018; Palomer et al., 2018
	Antagonist	GSK0660 GSK3787	Psoriasis	Induces anti-inflammatory property upon topical application	Hack et al., 2012
		FH535	Cancer	Introduces anti-proliferative activity in the cancer cells via inhibition of Wnt/β-catenin signaling pathway	Handeli and Simon, 2008
PPARG	Agonist	Prostaglandins	Cystic fibrosis Cancer Alzheimer’s disease and Parkinson’s disease	Inhibits the expression of *iNOS* thereby inhibiting the activation of Mϕ, reduced tissue damage due to less inflammatory responses 15d-PGJ_2_ restricts phosphoinositide 3-kinase (PI3K)-Akt pathway dependant cell proliferation of primary astrocytes, neuroblastoma, and carcinomas	Subbaramaiah et al., 2001; Rotondo and Davidson, 2002; Yagami et al., 2017
				Induces neuronal cell survival and neuro-protection	
		Thiazolidinediones (TZDs)	Type-2 diabetes Dyslipidemia Parkinson’s disease	Induces insulin sensitizing properties Balances extra- and intra-cellular lipid metabolism Profound expression of PPARG due to agonists lead to suppression of microglial activity thereby preventing neurodegeneration	Kadowaki, 2001; Chiarelli and Di Marzio, 2008; Connolly et al., 2015
		Fibrates	Dyslipidemia	Balances extra- and intra-cellular lipid metabolism	Gervois et al., 2004
		Unsaturated FA	High cholesterol Cardiovascular disease Hypertension	Alters membrane lipid concerto, cellular metabolism, and signal transduction	Bordoni et al., 2006; Villacorta et al., 2009; Kuna and Achinna, 2013; Banno et al., 2018
	Antagonist	GW9662 T0070907	Hematopoietic cancer	Induces anti-proliferative activity and apoptosis	Burton et al., 2008; Handeli and Simon, 2008

Induction of respiratory burst during mycobacterial infection is another important aspect to increase mycobactericidal activity of infected macrophages. The respiratory burst occurs due to induction of oxidative radicals, nitric oxide (NO) and superoxide ions after the phagocytosis of bacteria by macrophages. It has been reported that expression of PPARG leads to inhibition of p47 phagocyte oxidase (p47phox), an important component of NOX enzyme complex (Von Knethen and Brüne, 2018). This indicated that PPARG also has significant role in the regulation of ROS and NO production during infection process.

## Mycobacteria Regulate Oxidative Stress in Macrophages

**FIGURE 2:**
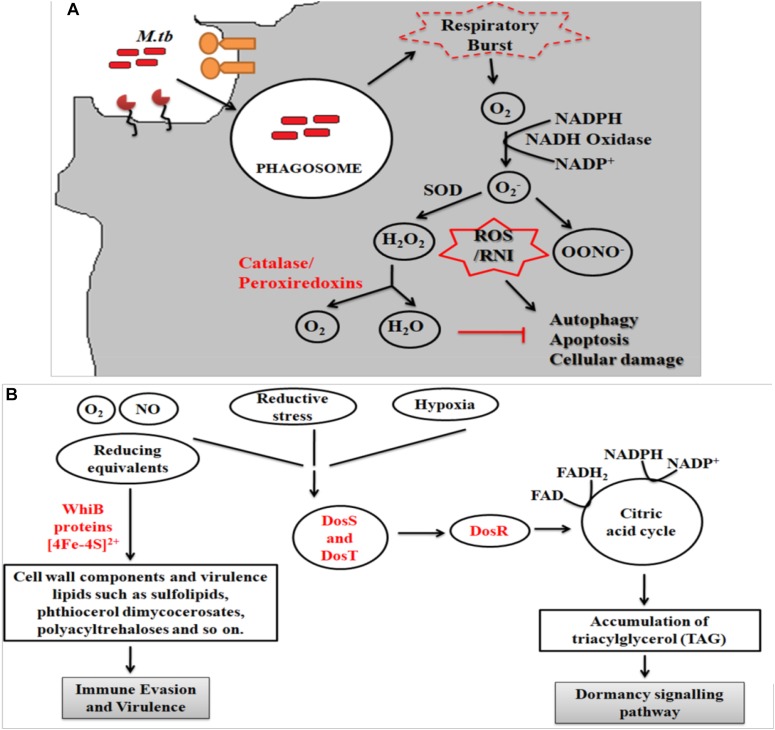
**(A)** Oxidative stress response. Following entry of *Mtb* into host cells results in the generation of oxidative stress responses. *Mtb* employs several strategies to combat these stress mechanisms, which includes activation of anti-oxidant enzymes (like superoxide dismutase, catalase/peroxidase, etc.), redox-sensors (like DosR regulon system) and redox buffering system. Phagocytosis of *Mtb* leads to formation of superoxide radical via NADPH oxidase. As evasion strategy, *Mtb* genes reduce the superoxide radical into less toxic intermediates using anti-oxidant enzymes. The pathway eventually inhibits the process of autophagy, apoptosis and cellular damage. **(B)** Mycobacterial oxidative stress response gene family protein, WhiB, combats the redox and nitrosative stress via the Fe–S clusters while DosS and T gene from DosR regulon senses the redox imbalance and activates the DosR to furtheractivate the dormancy pathway.

During infection *Mtb* successfully deals with a wide range of host immune responses. One of the major responses exerted by infected cells is the generation of oxidative radicals. These toxic radicals kill pathogens by causing disintegration of bacterial cell membrane, DNA damage, deactivation of key metabolic enzymes or proteins (Gupta and Chatterji, 2005; Dos Vultos et al., 2009; Nambi et al., 2015; Tyagi et al., 2015). Oxidative stress responses includes production of reactive oxygen species (ROS) and reactive nitrogen intermediates (RNIs) (Voskuil et al., 2011). However, pathogenic mycobacteria are able to inhibit oxidative stress mechanisms through modulation of different cell signaling mechanisms, up-regulation of anti-oxidant enzymes and redox buffering systems (Figure 2A) (Mohanty et al., 2015, 2016; Chao et al., 2017). NADPH oxidase 2 (NOX2) is the key enzyme responsible for the cellular ROS production by using superoxide radicals (${\text{O}}_{2}^{-}$) as precursor molecule (Lambeth, 2004). NOX is a multi-protein enzyme complex consisting of p40phox, p47phox, p67phox, p22phox, and gp91phox as core components. Alteration in any of these regulatory components compromises the function of NOX enzyme complex. NOX are recruited to the pathogen containing phagosomes to generate phagocytic oxidative stress and phagocytic burst to eliminate enclosed pathogen (Dan Dunn et al., 2015). NADPH oxidase generates superoxide radicals (${\text{O}}_{2}^{-}$), which are then converted to more toxic hydrogen peroxide (H_2_O_2_) in the presence of superoxide dismutase (SOD) and eventually reduced to water and molecular oxygen by catalase (Voskuil et al., 2011; Dan Dunn et al., 2015). *Mtb* is presumed to utilize ROS to cause genomic instability in the host cells by inducing excessive infiltration of immune cells in the lungs to cause lung lesions (Chao et al., 2017), and chromosomal instability (Mohanty et al., 2016).

The fate of ROS production depends upon the type of host pathogen interaction (Bruns and Stenger, 2014). It has been reported that interaction of *Mtb* with MR results in the down-regulation of ROS production by up-regulating the expression of anti-inflammatory cytokines and inhibiting the expression of pro-inflammatory cytokines like IL12 (Garcia-Aguilar et al., 2016). To counteract oxidative stress, PPARG modulates a wide range of anti- (like catalase) and pro-oxidant through NFκB transcription factor and other downstream signaling pathways (Girnun et al., 2002; Okuno et al., 2008; Polvani et al., 2012; Kim and Yang, 2013). These studies indicate that MR and PPARG are important for modulation of macrophage immune responses during mycobacterial infection. Different *Mtb* components such as glyco-conjugates (ManLAM), ESAT-6 like proteins, mycolic acids, ROS scavenging enzymes and regulatory proteins such as DosR regulon system are responsible for the development of ROS resistance (Voskuil et al., 2011). *Mtb* dormancy regulon system, DosR, is crucial for redox sensing. This regulon system mainly consists of three components- DosR, DosS, and DosT. Heme proteins DosS and DosT sense O_2_, NO, and CO and relays the signal to DosR to modulate the expression of genes responsible for energy production and utilization of host lipids to facilitate mycobacterial persistence. It is found that *Mtb* WhiB3 utilizes Fe–S cluster to respond to the exogenous or endogenous redox stress by utilizing the host fatty acid by β- fatty oxidation pathway to favor bacillary survival (Singh et al., 2009; Mehta et al., 2015) (Figure 2B). Several known mycobacterial enzymes and proteins that enable the bacilli to sense the redox imbalance has been enlisted in Table 2.

**Table 2:** List of known *Mycobacterium tuberculosis* oxidative stress response genes.

Gene name	Function	References
*Superoxide dismutase (Sod)c, Rv0432*	Reduces superoxide radicals to peroxide	Mehta et al., 2015
*SodA, Rv3846*	Reduces peroxide radicals to molecular oxygen and water	Mehta et al., 2015; Nambi et al., 2015
*DoxX*	Thiosulfate oxidation activity	Nambi et al., 2015
*DosR regulon (DosR, DosS, and DosT)*	Senses oxidative stress, combats hypoxic condition and dormancy	Leistikow et al., 2010; Nambi et al., 2015
*Rv3283 and RvSseA*	Thiosulfate oxidation activity	Nambi et al., 2015
*Catalase/peroxidise (KatG)*	Defense against reactive oxygen and nitrogen intermediates by reducing peroxide radicals to molecular oxygen and water	Milano et al., 2001; Bulatovic et al., 2002
*Thioredoxin reductase (Tpx), Rv1932*	Peroxide detoxification	Hu and Coates, 2009
*Alkylhydroperoxide reductase (AhpC and D)*	Offer defense against oxidative stress via NADH-dependent peroxidase and peroxynitrite reductase	Hillas et al., 2000; Springer et al., 2001; Lee et al., 2014
*Peroxiredoxin (AhpE)*	Peroxide detoxification	Jaeger, 2007; Perkins et al., 2015
*Rv3242c*	Inhibition of oxidative stress and autophagy pathway via MAPK pathway	Mohanty et al., 2015
*Rv2346c*	Higher intracellular survival by causing ROS dependant genomic instability	Mohanty et al., 2016
Succinate dehydrogenases complexes *(Rv0247c-Rv0249c)*	Modulates oxidative phosphorylation and central metabolism by maintaining membrane potential for energy production	Knapp et al., 2015; Nambi et al., 2015
*F420-dependent glucose-6-phosphate dehydrogenase, fgd*	Help in maintaining redox homeostasis and latency reactivation	Gurumurthy et al., 2013; Nguyen et al., 2017
*Rv1909c, FurA*	Transcriptional regulator of Katg gene	Milano et al., 2001; Eckelt et al., 2015; Nambi et al., 2015
*Rv1465*	Modulates cellular metabolism	Willemse et al., 2018
*Rv0035, fadD34*	Modulates fatty acid synthase thereby regulating oxidative metabolism	Agarwal et al., 2007; Mukhopadhyay et al., 2012
*noxR3*	Reactive nitrogen intermediate resistance gene inhibits the nitrostative stress inside the infected macrophages	Ruan et al., 1999
*whiB3, whiB 4 and whiB7*	Modulates redox homeostasis and lipid metabolism	Mehta et al., 2015; Nambi et al., 2015; Tyagi et al., 2015
*Rv2624c, Rv2026c*	Nucleotide-binding universal stress protein alters metabolic pathways via arginine in an ATP-dependent manner	Hingley-Wilson et al., 2010; Jia et al., 2016
*Rv1049, MosR*	Oxidation-sensing regulator, upregulates the expression of several oxidoreductases	Brugarolas et al., 2012
*Mtb sigma factor*	Stress-induced extracytoplasmic sigma factor, transcriptionally regulates the expression of different anti-oxidants	Raman et al., 2004
*Serine threonine kinases (Pkn family)*	Modulates acidic pH, hypoxia inside infected macrophages	Nambi et al., 2015; Venkatesan et al., 2016; Khan et al., 2017
*oxyR*	Oxidative stress regulators works in combination with aph and fur genes	Voskuil et al., 2011
*SoxR*	Manipulates the expression of both antioxidant genes and enzymes involved in the process of DNA repair which results in resistance toward oxidative stress and anti-bacterial activity of macrophage	Voskuil et al., 2011

The regulation of ROS production during mycobacterial infection is mainly studied in mitochondria, which are considered as primary source of cellular ROS production (Dan Dunn et al., 2015). Mitochondrial ROS (mtROS) is generated from oxidation of different metabolic intermediates produced during oxidative phosphorylation at the electron transport chain (ETC) system present in the inner mitochondrial space. Three complexes (Complexes I, II, and III) in the ETC play significant role in ROS generation. Electrons released during the conversion of NADH to NAD+ direct the partial reduction of oxygen to ${\text{O}}_{2}^{-}$. Approximately 80% of ${\text{O}}_{2}^{-}$ is released into the inter-membrane space of mitochondria and 20% remains in the matrix. The transition pore present in the mitochondria allows the release of ${\text{O}}_{2}^{-}$ into the cytoplasm, where it is dismutated to hydrogen peroxide (H_2_O_2_) in the presence of superoxide dismutase (SOD) (Dan Dunn et al., 2015). The role of mtROS has been well-studied with respect to cellular alterations in response to hypoxia, inflammation, autophagy, and cell differentiation processes (Voskuil et al., 2011). *Mtb* eis (enhanced intracellular survival) protein was found to inhibit JNK dependent ROS signaling by inducing acetylation of DUSP16/MKP-7, a JNK phosphatase. The acetylation of JNK phosphatase negatively regulates the autophagy process (Kim et al., 2012). A 38-kDa *Mtb* glycoprotein, PstS-1, was also reported to modulate oxidative stress signaling molecules to establish a successful intracellular infection (Esparza et al., 2015).

Besides mitochondria, another prime site for oxidative metabolism and redox homeostasis is the peroxisomes. Peroxisomes, a single membrane bound organelle of 0.1–0.5 μM size, are ubiquitously present in the cytoplasm of almost all eukaryotic cells. They are involved in the metabolism of long chain fatty acids, D-amino acids, polyamines, and the reduction of ROS (Schrader and Fahimi, 2006). Studies have shown that intricate inter-organelle communications between mitochondria and peroxisomes are very crucial for a broad range of cellular processes, including redox homeostasis mechanisms (Schumann and Subramani, 2008; Lismont et al., 2015). Peroxisomes are also associated with different human diseases. For example, deficiency of single peroxisomal enzymes like acyl CoA oxidases, adrenoleukodystrophy ALD gene and so on caused development of “empty” or non-functional peroxisomal membranes, known as “ghosts.” A mutation in adrenoleukodystrophy ALD gene, encoding for different peroxisomal ABC transporters, led to excessive accumulation of fatty acids which caused demyelination of the nervous system and death. Loss of peroxisome function has also been associated with cancer progression due to oxidative damage (Delille et al., 2006). The role of peroxisomes in various metabolic activities is largely dependent on its interaction with other subcellular organelle, majorly mitochondria. In the following section, we have discussed the interplay between mitochondria and peroxisome and its implications in cellular metabolism.

## Cross-Talk Between Mitochondria and Peroxisomes Are Essential for Cellular Metabolism

Mitochondria and peroxisomes are dynamic organelles present in all eukaryotic cell types (Demarquoy and Le Borgne, 2015; Lismont et al., 2015). Both organelles have direct implications on oxidative and fatty acid metabolism. Mitochondria and peroxisomes are derived from two different ancestors: mitochondria are derived from the endosymbiotic pathway, while peroxisome biogenesis is initiated from the endoplasmic reticulum (ER). However, both of them have been demonstrated to undertake metabolic cross-talk to maintain cellular homeostasis. For example, reoxidation of NADH to NAD^+^ generated during peroxisomal β-fatty acid oxidation occurs only after its interaction with mitochondria. Both peroxisomes and mitochondria follow common basic steps such as dehydrogenation, hydration, and thiolytic cleavage for fatty acid oxidation. However, the enzymes involved in catalyzing these reactions are different. In mitochondria, the initial step of fatty acid oxidation is catalyzed via FAD-dependant dehydrogenase, which directs the electrons toward ETC for ATP synthesis. In case of peroxisomes, FAD-dependant acyl-CoA oxidases catalyze the first step of β-fatty acid oxidation, where the electrons are targeted to the oxygen for the generation of superoxide ions. Unlike mitochondria, peroxisomes lack the respiratory chain, thus it can only metabolize the very long chain fatty acids (VLCFAs) into short chain fatty acids and acetyl-CoA that are then transported to the mitochondria as carnitine esters by different peroxisomal ABC transporters and aceyltransferases (Wanders, 2004). Import of fatty acids into the mitochondria is coupled with the generation of CO_2_, H_2_O, and ATP synthesis (Demarquoy and Le Borgne, 2015). Thus, we can speculate that the intracellular bacilli such as *Mtb* can meet their nutritional requirements by utilization of peroxisomal shorter chain fatty acids and the mitochondrial ATP molecules. These nutritional rich organelles thus may help to enhance bacillary persistence inside the host cells.

**FIGURE 3:**
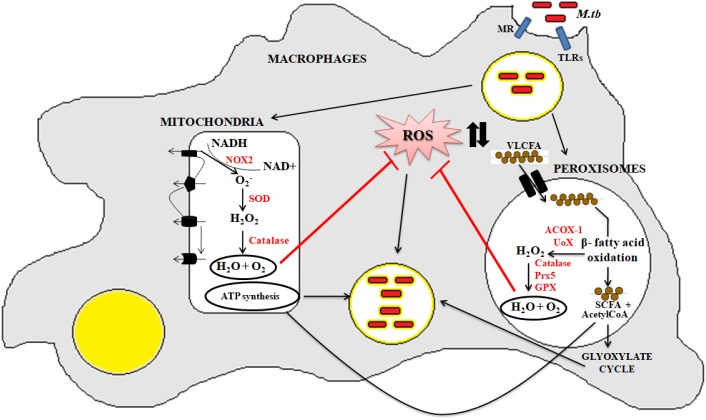
Crosstalk between mitochondria and peroxisome: the pathogenic *Mtb* enters into the macrophage cells via different PRRs like mannose receptor (MRs) or TLRs. After phagocytosis, *Mtb* modulates cellular oxidative stress mechanisms. Inside the mitochondria, NOX2 generates the superoxide radicals during the ETC cycle. These superoxide radicals are then metabolized to hydrogen peroxide by superoxide dismutase. Anti-oxidant enzyme catalase eventually breaks down the toxic H_2_O_2_ into water and molecular oxygen. This phenomenan helps to increase the bacillary count inside the cells. In peroxisomes, the bacteria metabolize the stored fatty acids and lipids for its survival. During the peroxisomal β- fatty acid oxidation, acyl CoA oxidases, and other peroxisomal oxidases metabolizes the very long chain fatty acids (VLCFA) into short chain fatty acids (SCHFA) and acetylCoA. The SCHFA is exported into the mitochondria for further metabolism to generate ATP. The acetyl CoA is used as an intermediate molecule in the glyoxylate shunt pathway. In addition to generation of SCHFA, cellular ROS is formed which is further catalyzed by the array of anti-oxidant enzymes in peroxisomes like catalase, glutathione peroxidise (GPX), and so on. This phenomenon further provides a favorable niche for the bacilli to survive.

Peroxisomes have a complex array of pro-and anti-oxidant system such as catalase, glutathione peroxidase, SOD, and so on. It has been speculated that the toxic H_2_O_2_ acts as a key messenger molecule for peroxisome function in maintaining cellular redox homeostasis, β-fatty acid oxidation, lipid metabolism, and induction of innate immunity against pathogens (del Río, 2011; del Río and López-Huertas, 2016) (Figure 3). For survival, intracellular pathogens need to attain metabolic adaptation to counteract the oxidative stress generated during the host pathogen interaction. In this context, *Mtb* WhiB3 protein was found to modulate the host fatty acid and lipid metabolism in response to oxido-reductive stress to maintain the intracellular redox balance. It was reported that WhiB3 acts like a redox sensor to maintain redox balance and innate immunity (Singh et al., 2009). Unlike mitochondria, the oxidative metabolism occurring inside the peroxisomes is not coupled to oxidative phosphorylation. Thus instead of ATP formation as is the case in mitochondria, the free energy generated in peroxisomes is released as heat inside the cells (Schrader and Fahimi, 2006).

## Peroxisomal Biogenesis and Its Role During Infection

Peroxisomes have been found to dampen macrophage activation and also mediate immunomodulatory functions. In lung inflammatory diseases, like cystic fibrosis, peroxisomes dampen LPS-induced pro-inflammatory proteins like COX2, TNFA, and IL6 and so on. In addition, activation of PPARs have been shown to transactivate genes involved in the functioning of peroxisomes via transrepression of inflammatory response (Girnun et al., 2002; Di Cesare Mannelli et al., 2014; Vijayan et al., 2017). Thus we can speculate that immune regulatory role of peroxisome may aid bacillary persistence in latency due to its role in later phase of inflammation.

**FIGURE 4:**
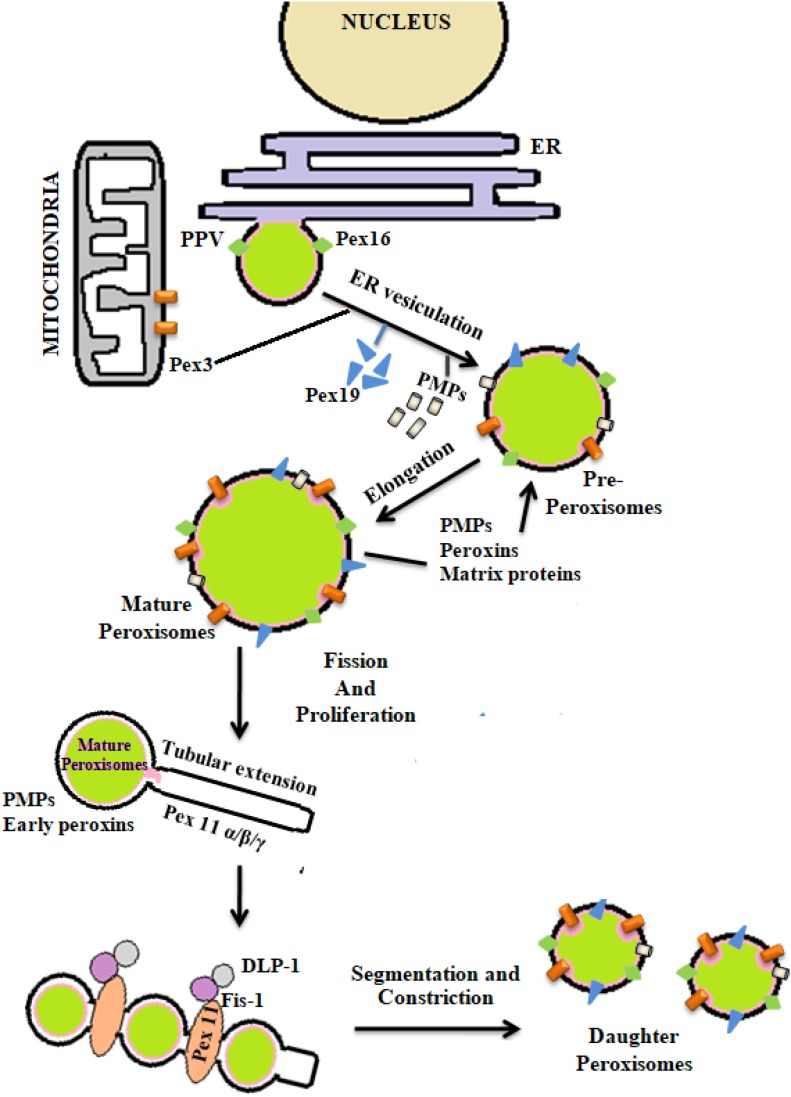
*De novo* biogenesis of peroxisomes: peroxisomes originate from endoplasmic reticulum (ER) and mitochondria. With the help of different peroxins like PEX3, 16, 19, and also peroxisomal membrane proteins (PMPs) active organelle is formed. Balance in cellular oxidative metabolism results in balanced redox interplay between pro- and anti-oxidants of peroxisomes thereby helping in the proper growth and division of daughter peroxisomes from pre-existing peroxisomes via the expression of PEX11, DLP-1, Fis, and Mff.

The process of peroxisomal biogenesis is still poorly understood. There were two different models of peroxisome biogenesis which have co-existed for years. One in which peroxisomes arise from the pre-existing peroxisomes acquiring different PMPs and matrix peroxin proteins thereby dividing into daughter peroxisomes via fission, known as growth division model. The second being *de novo* biogenesis, where the membrane proteins are introduced into the ER membrane, further imported to the preperoxisomal ER (pER) region, from where distinct pre-peroxisomal vesicles (ppVs) originate. The ppVs containing different PMPs fuse with the pre-existing peroxisomes to create mature peroxisomes. Lately a third model has been envisioned which blends both the growth and division models with *de novo* biogenesis models (Farré et al., 2019). In general peroxisome biogenesis involves three major steps- (i) arrangement of peroxisomal membrane, (ii) import of proteins to the peroxisomal membrane, and (iii) maturation of the organelles (Eckert and Erdmann, 2003). Few reports have demonstrated peroxisome dense-areas in the proximity of ER and thus proposed that the peroxisomes are formed primarily by a “ER vesiculation” process. This pathway has been well-studied in the yeast system and is still considered as the most common pathway. The biogenesis of peroxisomes involve several peroxins (PEX) and peroxisomal membrane proteins (PMPs) (Titorenko and Mullen, 2006). The peroxins such as PEX16, PEX3, and PEX19 are involved in initial steps of peroxisomal membrane biogenesis and transport of other peroxins into the peroxisomes after post-translational modifications. It was reported that the PEX16 and PMPs are the first to enter the ER via translocons, followed by their recruitment to the exit site of the ER. The PMPs and PEX16 exit the ER in the form of buds to which mitochondria derived PEX3 are recruited to form the pre-peroxisomal vesicles (PPV). Maturation of the pre-peroxisomal vacuole into a metabolically active peroxisome organelle is further dependant on other peroxins such as PEX19, 14, 11, 5, matrix proteins and PMPs which are targeted either via PTS (Peroxisomal target signaling)-1 or 2 (Agrawal and Subramani, 2016) (Figure 4). PTS binds to the cytosolic receptors of the peroxisomes such as PTS-1, which then interacts with PEX7p. This pathway is most commonly seen in yeast system. In case of mammalian cells, PTS-2-PEX5p interaction is more prevalent and well-studied. These membrane proteins and docking factors subsequently initiate the translocation of other peroxisomal proteins like PEX8p, PEX10p, PEX20p, PEX1p, PEX2p, PEX4p, PEX6p, PEX17p, and PEX22p for successful peroxisome biosynthesis (Collins et al., 2000; Brown and Baker, 2003). Once the mature peroxisomes are formed, different isoforms of PEX11p initiate the process of segmentation and constriction to form new daughter peroxisomes. Elongation, constriction, and fission of peroxisomes to form new peroxisomes is significantly dependant on the expression of PEX11B, dynamin-like protein 1 (DLP1), mitochondrial fission factor (Mff), and Fission 1 (Fis1) (Lingard and Trelease, 2006; Itoyama et al., 2013) (Figure 4). PEX11 proteins initiate the preliminary step(s) of peroxisomal division and proliferation by membrane reorganization. PEX11 proteins then assemble the other division machineries DLP-1, Fis1, and Mff. Fis1 and Mff promote the recruitment of DLP-1 to the mammalian peroxisomes (Schrader et al., 2012). DLP-1 supports the maintenance of peroxisomal morphology throughout the process of membrane fission via formation of large multimeric spirals. This pathway is well-studied in the yeast system; however the precise order in which these peroxins act is not clearly understood. To understand the mechanism of peroxisome biosynthesis in response to bacterial infection, recently our group has provided some evidences that *Mtb* putative mannosylated acetyltransferase triggers peroxisome biogenesis through ER vesiculation process in macrophages. We observed that *Mtb* acetyltransferase induce the expression of PEX11, PEX19, PEX5 and peroxisomal membrane proteins 70 (PMP70) in infected macrophages. Peroxisomes are known as an important organelle in the maintenance of ROS/RNS homeostasis with the help of H_2_O_2_ producing and degrading enzymes, oxidases, and catalase present in the peroxisomes (Bonetta, 2005). In addition, peroxisomes also play an important role in the induction of innate immune responses during viral and bacterial infections (Lazarow, 2011; Odendall and Kagan, 2013; Boncompain et al., 2014). These studies have shown that innate immune receptors such as RIG-I-like Receptor (RLR) proteins determines the fate of infection in human cells by inducing the expression of different forms of interferons (IFNs) that were majorly found in peroxisomes (Dixit et al., 2010; Odendall and Kagan, 2013; Pandey et al., 2014). The interaction between innate immune receptors and IFNs in response to intracellular infection activated the Janus kinases/signal transducer and transcription activator (JAK/STAT) pathway (Bordon, 2014). *Mtb* infection is known to modulate these pathways to favor its persistence in macrophages. Inhibition of JAK/STAT signaling pathway reduced intracellular mycobacterial burden due to alteration in the expression of different transcription factors and delayed immune response in infected macrophages (Yuhas et al., 2009; Péan et al., 2017). This provides a hint that peroxisomes may also have a crucial role in the determination of mycobacterial survival inside the host cells. However, further investigation is required to interlink these signaling cascades with peroxisomes in determining the fate of *Mtb* infection.

## Peroxisomes Shield Intracellular Pathogens From Oxidative Stress

So far the functions of peroxisomes have been extensively studied in plants and yeast, where few peroxisomal enzymes such as acyl-CoA oxidases and urate oxidase were shown to produce H_2_O_2_ as part of their metabolic activities during peroxisomal β-fatty acid oxidation process. To counter balance this rise in endogenous ROS level, peroxisomal catalase converts the toxic H_2_O_2_ into water and molecular oxygen. Catalase, a porphyrin heme containing enzyme plays an important defensive function in protecting the organelle from the adverse effects of accumulating peroxides, is targeted to the peroxisomes via PEX5 protein (Fransen et al., 2012). However, this system has not been clearly defined in mammalian cells and remains largely unknown. We have recently shown that *Mtb* enters macrophages via mannose receptors (MRs), which then leads to the activation of PPARG nuclear receptor. The up-regulation of PPARG was found to regulate the synthesis of peroxisomal oxidative and anti-oxidative enzymes like urate oxidase, acyl-CoA oxidase, and catalase thus inhibition of cellular ROS. *Mtb* infection induces the expression of global transcriptional regulator via some novel effector molecules belonging to the Bcl-2 family members like pro-apoptotic, Bax and pro-survival, Mcl-1 proteins. Thus PPARG limits the important defense mechanism, apoptosis, during *Mtb* infection (Arnett et al., 2018). Similarly another study has shown that induction of PPARG increased the synthesis of catalase through PPARG response element, thereby resulting in the reduction of oxidative stress (Okuno et al., 2010; Khoo et al., 2013; Di Cesare Mannelli et al., 2014). Another study found that inhibition of ROS production by PPARG is dependent on redox-sensitive NFκB and HIF1A transcriptional factors in C57BL/6 mouse model (Lee et al., 2006). These studies provide sufficient evidences that peroxisomes are critical organelles in the regulation of oxidative stress levels produced in response to different stimuli. These evidences also suggest that intracellular pathogens, including *Mtb* may hijack peroxisomes to turn the intracellular microenvironment in favor of pathogens.

## Peroxisomes Regulate Innate Immunity to Counteract Infection Process

*Mtb* contains plethora of virulence factors that modulate host immune responses, including ROS dependant signaling cascades to create a favorable niche for bacteria. One of such pathways altered during oxidative metabolism is the autophagy mechanism (Mohanty et al., 2015; Awuh and Flo, 2017). Autophagy is a dynamic self-degradative process known to regulate the expression of different pro- and anti-inflammatory cytokines and subsequently downstream signaling molecules. Autophagy is classified into three major subgroups: chaperone mediated autophagy (CMA), microautophagy, and macroautophagy (Mizushima, 2007; Glick et al., 2010). Both macro and micro-autophagy are able to engulf large structures through selective and non-selective mechanisms. In selective degradation, specific target molecules such as damaged or unused organelles and pathogens are targeted to the autophagosome, however in case of non-selective degradation, any random cargo like peptides are degraded (Glick et al., 2010). In CMA targeted proteins are translocated across the lysosomal membrane in a complex with chaperons such as HSP-70/co-chaperons. The substrates are identified in the cytosol through the binding motif and translocated into the autophagosome (Glick et al., 2010; Kaushik and Cuervo, 2012; Cuervo and Wong, 2014). *Mtb* deploy different virulence factors to inhibit redox dependent autophagy mechanism to aid its persistence in macrophages (Shin et al., 2010; Kleinnijenhuis et al., 2011; Kim et al., 2012; Mohanty et al., 2015). The first adapter molecules involved in the autophagy process are SQSTM1/p62, NBR1, NDP52, and NIX (Johansen and Lamark, 2011; Behrends and Fulda, 2012; Lippai and Löw, 2014). These receptors contain LC3 (microtubule-associated protein 1 light chain 3) interacting regions, and can therefore directly bind to LC3, which is a hallmark protein of autophagy. During the autophagic flux LC3-I is delipidated to LC3-II, a marker protein for autophagy induction. Increased LC3-II puncta in autophagosomes along with autophagy related genes (*Atgs*) and *beclin* encourages its fusion with lysosome to form autophagolysosomes. Inside the autophagolysosome the unwanted peptides, microorganisms and unused cellular organelles are degraded to maintain cellular balance.

**FIGURE 5:**
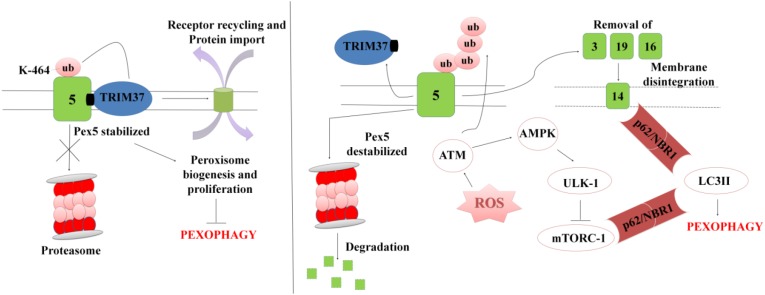
A representation for Pexophagy: PEX5 is stabilized by the mono-ubiquitination at K464 position via TRIM37, which further helps in maintaining the peroxisome homeostasis. In response to peroxisomal ROS, PEX5 undergoes polyubiquitination via activation of ATM-kinase. PEX5 is degraded via proteosome lysis thereby inducing Pexophagy via activation of autophagy adaptor molecules SQSTM1/p62 and NBR1. Degradation of PEX5, leads to peroxisomal disintegration and removal of PEX3 and other peroxins. Removal of PEX3 initiates peroxisome sequestration via activation of PEX14, which acts as a docking site for LC3II. The interaction eventually leads to degradation of bulky and damaged organelles via Pexophagy.

In order to maintain cellular homeostasis it is important to maintain the rate of peroxisomal degradation and biogenesis events. The event is regulated by the intracellular metabolic signaling and nutrient availability. To maintain the turnover the damaged or excessive peroxisomes are degraded via selective autophagic pathway known as “Pexophagy,” shown in Figure 5 (Wang et al., 2015; Tripathi et al., 2016). The first molecule to respond to peroxisomal ROS is an ataxia-telangiectasia mutated (ATM) kinase, which acts as an apical activator during DNA damage response (Tripathi et al., 2016). The target molecule is transported into the peroxisomes via PEX5, which then activates TSC2 (tuberous sclerosis complex 2) leading to inhibition mTORC expression. The mTOR inhibition in response to ROS co-regulates the expression of other peroxins. During coordinated transition between peroxisome biogenesis and degradration, PEX5 undergoes mono-ubiquitination via TRIM37, an E3 Ligase. Monoubiquitination of PEX5 is essential for import of peroxisomal matrix proteins into the peroxisomes. However with mutations at TRIM37 or its removal as a result of external stimulus, like excessive ROS formation, destabilizes PEX5 and it undergoes rapid degradation due to poly-ubiquitination that eventually interfere with the receptor recycling leading to damaged or bulky peroxisomes (Wang and Subramani, 2017; Wang et al., 2017; Cai et al., 2018). Degradation of PEX5 results in sequestration of dysfunctional peroxisomes, which are further recognized by autophagy adaptor molecules p62 and NBR1. Initiation of Pexophagy also results from the removal of peroxisomal integral membrane protein PEX3. Removal of PEX3 activates the docking sites of LC3-II in PEX14 (Bellu et al., 2002). Reports also state that inhibition of mTORC leads to active translocation of transcription factor EB (TFEB) into the nucleus which eventually increases autophagic flux and the expression of LC3-II, ATGs, and Beclin via interaction of the ubiquitinated proteins with SQSTM1/p62 and NBR1 (Tripathi et al., 2016). In order to maintain metabolic equilibrium, in addition to regulate peroxisome turnover it is important to regulate β-oxidation of long chain fatty acids and detoxification of oxidative radicals in peroxisomes. To achieve this, peroxisomes avoid Pexophagy pathway and rather undergo growth and division from pre-existing peroxisomes to form new functional peroxisomes. Our recent unpublished data has shown that *Mtb* acetyltransferase plays a role in the maintenance of peroxisome turnover via the formation of daughter peroxisomes from pre-existing mature peroxisomes to scavenge cellular ROS formation. We observed that induction of ROS production and autophagy after serum starvation, zymosan and rapamycin treatment induced the Pexophagy mechanism in macrophages. Our data also showed that exposure to external stress conditions lead to oxidative damage resulting in the formation of damaged peroxisomes, which were then degraded to maintain metabolic balance inside the cells. We concluded that occurrence of these events in response to *Mtb* infection helps in mycobacerial persistence in infected cells.

## Peroxisomes: *hub* for Fatty Acid Metabolism

In addition to redox homeostasis, peroxisomes are also responsible for lipid metabolism and β-fatty acid oxidation (Poirier et al., 2006). The PPARs (especially PPARA and G) significantly contribute to the lipid and carbohydrate metabolism, and also in the regulation of host inflammatory activities in response to various stimuli. PPARA is known to induce the expression of acyl CoA oxidase (ACOX1), enoyl coenzyme A hydratase 1 (ECH1), peroxisomal thioloases ACCA1 and 2 and PEX11A (Mandard et al., 2004; Rakhshandehroo et al., 2010). These molecules are involved in the fatty acid oxidation and peroxisome turnover in hepatocytes (Dan Dunn et al., 2015). However, there are only few evidences available that extensively demonstrate the role of PPARA as metabolic regulator with respect to maintenance of cellular homeostasis during mycobacterial infection (Kim et al., 2017).

For many pathogens the establishment and progression of the disease largely depends upon the availability of nutrients. Few reports demonstrate that *Mtb* is able to utilize host derived nutrients to support its growth inside the host cells (Lee et al., 2013). *Mtb* as well as other intracellular pathogens oxidize saturated fatty acids to acetylCoA that is utilized to generate ATP through other metabolic pathways (Lee et al., 2013; Longo et al., 2016; Adeva-Andany et al., 2018), and the short chain fatty acids, which are further metabolized for energy production via β-fatty acid oxidation and glyoxylate shunt cycle (Williams et al., 2011; Toledo and Benach, 2015). *Mtb* has also been shown to utilize host fatty acids to combat metabolic stresses such as generation of toxic intermediates after propionyl-CoA metabolism (Lee et al., 2013). Several *Mtb whiB* genes (*whiB1–whiB7*) counteract the oxidative stress generated during the metabolism of fatty acids through β-fatty acid oxidation pathway (Kumar et al., 2011). Degradation of straight chain saturated fatty acids in peroxisomes requires participation of four enzymes namely Acyl-CoA oxidase (similar to Acetyl-CoA dehydrogenase in mitochondria), Enoyl-CoA hydratases, 3-hydroxyacyl-CoA-dehydrogenase, and 3-ketoacyl-CoA thiolase. Peroxisomes use a slightly modified oxidation process to shorten VLCFA to short chain fatty acids that can then be transported to the mitochondria to complete oxidation process (Poirier et al., 2006; Kretschmer et al., 2012). In case of mitochondria, the shorter chain fatty acids enters the electron transport via Acetyl-CoA dehydrogenase, which eventually leads to ATP production, however in case of peroxisomal β-fatty acid oxidation presence of different oxidases leads to formation of ROS. On contrary, disruption of peroxisomal β-fatty acid oxidation process using thioridazine hydrochloride inhibitor resulted in reduction of *Mtb* survival due to increase in total ROS production in infected macrophages. In addition to β-fatty acid oxidation, the role of peroxisomes in lipid metabolism has also been studied. We showed that *Mtb* infection induce PPARG dependent expression of different peroxins and enzymes involved in β-fatty acid oxidation and lipid metabolism. Various enzymes such as Diacylglycerol *O*-Acyltransferase 2 (DGAT2), 1-acylglycerol-3-phosphate *O*-acyltransferase 9 (AGPAT9*)*, Acyl-coenzyme A thioesterase 11 *(*ACOT11*)*, fatty acid synthase (FASN*)* were found to be important for lipid metabolism (Duszka et al., 2017). The lipids stored inside the host cells are basically used by the pathogens as a potential source of energy especially under serum starved conditions. A cross-talk between ER derived lipid droplets and peroxisomes have been studied extensively in yeast system, however its precise role during mycobacterial infection is unclear. It is presumed that the involvement of peroxisomes in the metabolism of host derived long chain and polyunsaturated fatty acids would support mycobacerial survival and growth.

## Conclusion and Future Perspectives

Intracellular pathogens such as *Mtb* are known to employ several strategies to suppress oxidative stress mechanisms to avoid killing by host cells. Different PRRs and PAMPs participate in the initiation of host–pathogen interactions, which subsequently result in the activation of various downstream signaling pathways and nuclear transcription factors such as PPARG and NFκB. These transcription factors regulate the expression of different pro- and anti-oxidants in mitochondria and peroxisomes to either eliminate or control the bacterial burden. In eukaryotic cells, mitochondria, and peroxisomes are the primary sites responsible for the maintenance of redox balance. Although, the role of mitochondrial ROS in redox homeostasis and innate immunity is well-defined, the presence of a set of pro- and anti-oxidants in the peroxisomes was also found important during infection process. Peroxisomes act as an important link between metabolic network and oxidative metabolism during bacterial infection. It is presumed that peroxisomes not only facilitate the maintenance of redox balance, but also provide a favorable niche for the bacterial survival by providing host-derived fatty acids and stored lipids as nutritional sources.

Very successful pathogens like *Mtb* require novel therapeutic interventions. So far, administration of antibacterial drugs were only considered as most popular method of treatment, however, abuse of these drugs for decades evolutionarily resulted in the development of antimicrobial resistance. This is posing serious threats to the healthcare system globally. To overcome these challenges, development of host-directed therapies that target the potential effector signaling molecules or boosting the cellular immunity are now considered as important milestones in the development of an adjunct therapy for the effective treatment of many notorious infections such as tuberculosis (Hancock et al., 2012). In this context, use of immunotherapy involving cytokines, antibodies, and nuclear transcription factors such as PPARs are being considered for the treatment of various diseases. PPARG agonists or antagonists have already been explored and demonstrated to reduce the antibiotic dosages and also improved the bacillary clearance in chronic granulomatous diseases (Terlecky et al., 2012; Reddy et al., 2016). In case of *Streptococcus pneumoniae* infection administration of PPARG ligand, ciglitazone, was found to alleviate lung inflammation (Subbaramaiah et al., 2001; Banno et al., 2018), thus targeting the expression of ligand would probably reduce lung inflammation by killing the bacteria. Targeting PPARs would also be beneficial for the host as it is known to modulate the expression of NFκB, which is a key regulator of several pro-inflammatory cytokines that are important for the augmentation of innate immune responses. Respiratory burst is another crucial phenomenon occurring during mycobacterial infection. It occurs due to accumulation of VLCFAs, which results in mitochondrial dysfunction and production of higher oxidative stress (Shi et al., 2012). This is proved to be detrimental for the bacillary persistence. Interference in peroxisome functionality using phenothiazine drug, thioridazine, resulted in accumulation of VLCFAs, thereby leading to the production of toxic intermediates of redox metabolism. These toxic radicals were shown helpful in the bacterial killing (Van den Branden and Roels, 1985). Considering the fact that *Mtb* prolongs its survival by utilizing host-derived nutrients through glyoxylate shunt pathway, it is worth to explore the treatment regimens that target glyoxylate intermediates to impair mycobacterial infection process. Inactivation of glycoxylate shunt pathway key enzymes such as isocitrate lyase and malate synthase may result in reduced uptake and metabolosis of the imported fatty acids and ATP synthesis, which eventually may make *Mtb* nutritionally deprived (Ahn et al., 2016). Thus development of combinatorial host directed therapies involving manipulation of cellular signaling intermediates, alteration of innate immune receptors and interference in the metabolic activities of *Mtb* may lead to potential therapeutic strategies for the treatment of drug resistant tuberculosis.

## Author Contributions

GG and UM wrote the manuscript. AS wrote the manuscript, designed content, and provided resources.

## Conflict of Interest Statement

The authors declare that the research was conducted in the absence of any commercial or financial relationships that could be construed as a potential conflict of interest.
